# Development of postoperative bronchopleural fistula after neoadjuvant immunochemotherapy in non-small cell lung cancer: case reports and review of the literature

**DOI:** 10.1007/s00432-024-05683-9

**Published:** 2024-04-04

**Authors:** Renshan Zhao, Xiaomin Guan, Peng Zhang, Yunpeng Liu, Yinghui Xu, Chao Sun, Shi Qiu, Wenhao Zhu, Zhiguang Yang, Xu Wang

**Affiliations:** 1 Cancer Center, The First Hospital of Jilin University, 1 Xinmin Street, Changchun, 130021 Jilin China; 2 Thoracic Surgery Department, The First Hospital of Jilin University, 1 Xinmin Street, Changchun, 130021 Jilin China

**Keywords:** Immune checkpoint inhibitors, Non-small cell lung cancer, Neoadjuvant therapy, Immune-related adverse event, Bronchopleural fistula

## Abstract

**Background:**

The advent of immune checkpoint inhibitors has dramatically changed the treatment paradigm for advanced non-small-cell lung cancer (NSCLC). Due to the complexity and diversity of stage III disease, the inclusion of immune checkpoint inhibitors (ICIs) in neoadjuvant treatment regimens is also required. However, immune-related adverse events (irAEs) limit the application of ICIs to a certain extent. Bronchopleural fistula (BPF) is a serious and fatal complication after pneumonectomy that is rarely reported, especially in patients who accept neoadjuvant immunotherapy or chemoimmunotherapy.

**Case presentation:**

Herein, we reported four patients with postoperative BPF who received a neoadjuvant regimen of sintilimab plus chemotherapy. Postoperative BPF occurred in the late stage in three patients; one patient underwent bronchoscopic fistula repair, and the fistula was closed well after surgery, and the other two patients gradually recovered within 1–2 months after symptomatic treatment with antibiotics. One patient with BPF after left pneumonectomy died of respiratory failure due to pulmonary infection. We also reviewed the literature on the development of postoperative BPF in patients receiving immuno-neoadjuvant therapy to discuss the clinical process further, postoperative pathological changes, as well as risk factors of BPF patients.

**Conclusions:**

Central type lung cancer with stage III may be the risk factors of BPF in cases of neoadjuvant immunochemotherapy for lung cancers patients.

## Introduction

Immune checkpoint inhibitors (ICIs) have dramatically changed treatment paradigms and outcomes for patients with advanced NSCLC (Doroshow et al. 2019). A pilot study published in the *New England Journal of Medicine* shed new light on neoadjuvant nivolumab in resectable early (stage I, II, or IIIA) NSCLC. Besides, the NADIM study reported that neoadjuvant therapy combined with immunotherapy could significantly increase the MPR (83%) of patients with stage III NSCLC (Provencio et al. 2020).

Therefore, it is of utmost importance to understand the role of immunotherapy in neoadjuvant therapy. A special attention should be paid to treatment-related adverse events. A number of isolated case reports have demonstrated that postoperative bronchopleural fistula (BPF) following lobectomy after neoadjuvant immunotherapy or chemoimmunotherapy may complicate immune checkpoint inhibitor therapy (Cao et al. 2020; Menezes et al. 2020; Cascone et al. 2021; Dai et al. 2022). However, there are still no reported series. Thus, the timing and spectrum of BPF are poorly understood. In this report, we presented four patients with BPF after neoadjuvant immunochemotherapy diagnosed at the First Hospital of Ji Lin University. We next conducted a comprehensive review of the related literature to further describe the characteristics of postoperative BPF occurring in NSCLC patients under neoadjuvant immunotherapy or immunochemotherapy and discuss the clinical process, pathological changes, as well as risk factors of BPF.

## Case presentation

This retrospective case study included patients with postoperative BPF associated with neoadjuvant immunochemotherapy (sintilimab plus nab-paclitaxel and carboplatin) who were admitted to the First Bethune Hospital of Jilin University in Changchun, China, between 2 December 2019 and 28 February 2021. TNM staging was according to American Joint Committee on Cancer (AJCC) 8th edition. The tumor response was assessed according to the Response Evaluation Criteria in Solid Tumors (RECIST) 1.1 version. The adverse events (Aes) were evaluated according to Common Terminology Criteria for Adverse Events (CTCAE) Version 5.0. Data were retrieved from electronic medical records, including demographics, clinical characteristics, laboratory data, pathology findings, and imaging images. Two medical oncologists (Xu Wang and Chao Sun) independently reviewed the data.

The study was approved by the Ethics Committee of The First Hospital of Jilin University in Changchun, China. Four patients developed BPF after surgery; one patient died of respiratory failure due to pulmonary infection, one patient underwent bronchoscopic fistula repair, and the fistula closed well after surgery, and the other two patients recovered gradually within 3–6 months after symptomatic treatment with antibiotics.

## Case 1

**Fig. 1:**
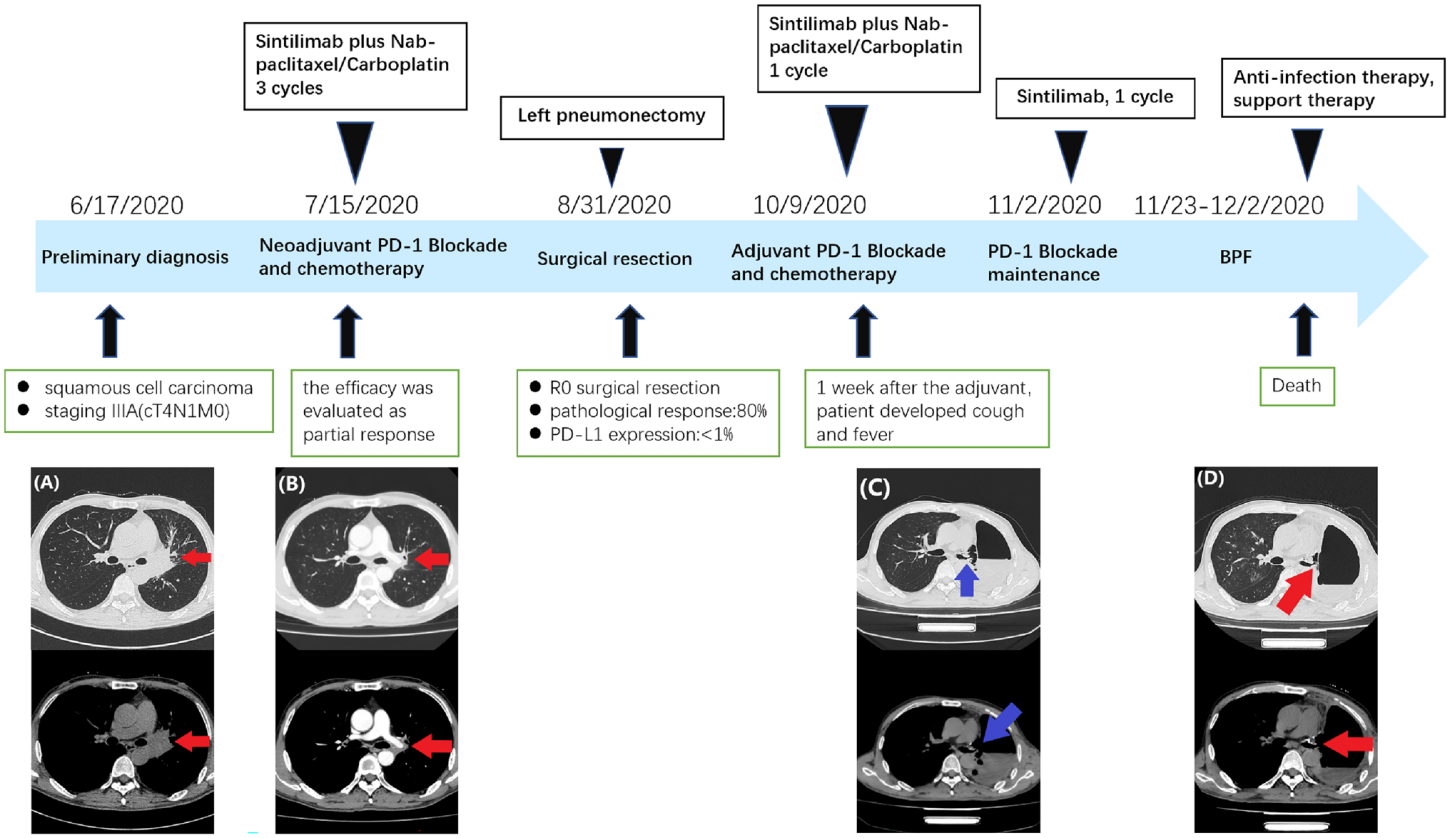
The timeline of the patient’s treatment course and evaluation of imaging efficacy. **A** Chest CT image before neoadjuvant therapy on 22 June 2020 shows a high-density shadow on the left hilum, about 4.0 × 2.9 cm in size (arrow). **B** Chest CT image after three cycles of neoadjuvant therapy on 25 August 2020 shows that the left hilar mass was significantly reduced compared to before (arrow). **C** Chest CT image 6 weeks after left pneumonectomy on 9 October 2020. On 24 November 2020, we reviewed the patient’s previous lung CT (9 October 2020), and there was a small fistula between the bronchial anastomosis and the chest cavity (arrow). **D** Chest CT image on 24 November 2020 shows that the fistulous tract had no obvious changes (arrow)

**Fig. 2:**
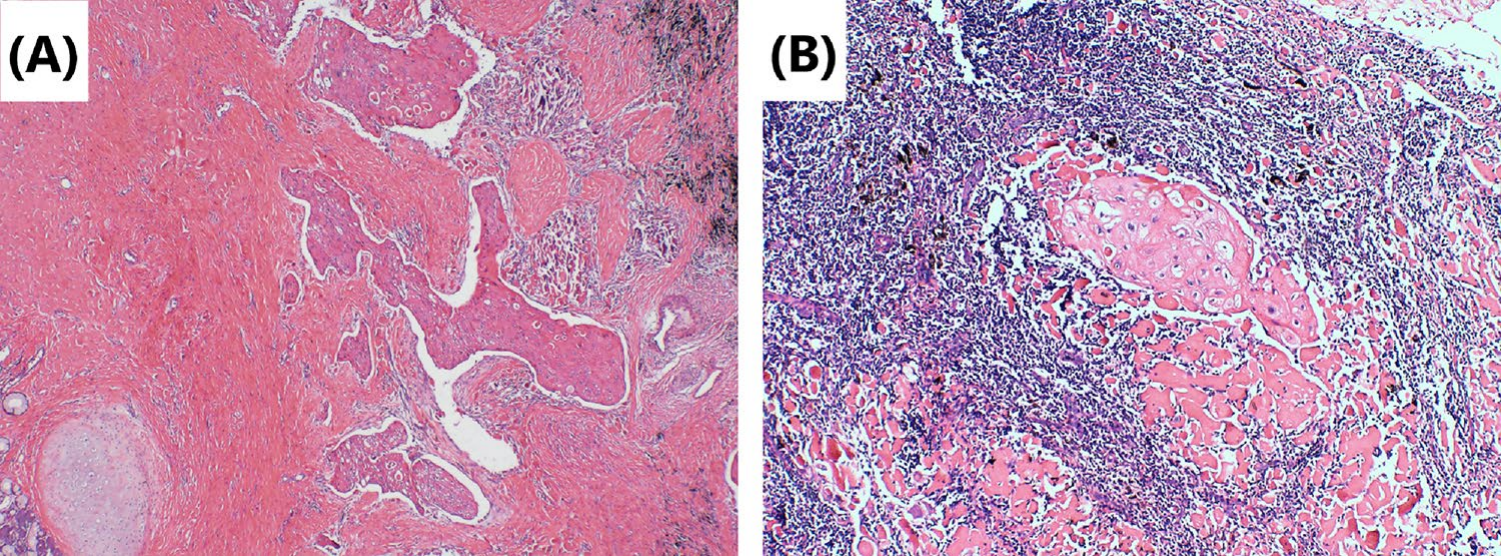
Tumor cells’ morphology is shown by hematoxylin–eosin staining. **A** Repairing changes are seen after neoadjuvant immunochemotherapy. Significant proliferation of fibrous tissue around the cancer nest with a small amount of chronic inflammatory cell infiltration. Original magnification × 40. **B** Lymph-node metastases are mainly repairing changes after neoadjuvant immunochemotherapy. Fibrous tissue hyperplasia around the cancer nest. Original magnification × 100

In June 2020, a 48-year-old male patient was diagnosed with stage IIIA (cT4N1M0) squamous cell lung carcinoma. The timeline of the patient’s treatment course and imaging response assessment are shown in detail in Fig. 1. The patient underwent 3 cycles of neoadjuvant immunochemotherapy and stopped medication 25 days prior to radical surgery. The therapeutic effect was evaluated as partial response (PR). Left pneumonectomy was performed and the postoperative pathological changes are shown in Fig. 2. The postoperative chest CT (9 October 2020) showed no obvious abnormalities (Fig. 1C1), so adjuvant immunochemotherapy was performed 41 days after the operation. Three days after the first course of adjuvant treatment, the patient developed transient fever (highest temperature was 38.6 ℃, grade 1), which subsided after only a few hours without other symptoms, and it did not occur before receiving the next cycle of adjuvant therapy. Considering that the patient has only the right lung reserve, the multidisciplinary team recommended single sintilimab maintenance treatment after performing the evaluation. Approximately 2 weeks after one cycle of sintilimab treatment, the patient developed persistent fever (highest temperature was 39.4 ℃, grade 2), cough, sputum, and dyspnea. Re-examination of chest computed tomography (CT) showed striped liquid–vapor density shadows on the left side of the thoracic cavity, and an obvious fistula was seen (Fig. 1D). Next, we reviewed the patient’s previous chest CT (9 October 2020), detecting a small fistula that was not easily found between the bronchial anastomosis and the chest cavity (Fig. 1C2). The patient received anti-infection and antifungal treatment for 1 week, but refused respiratory support and additional therapeutic intervention, such as bronchoscopy and fistula repair. As a result, his dyspnea progressively worsened, and the partial pressure of oxygen was only 65% under the state of oxygen inhalation. Due to the refusal of respiratory support, his partial pressure of oxygen progressively decreased. Unfortunately, the patient died on 2 December 2020.

## Case 2

**Fig. 3:**
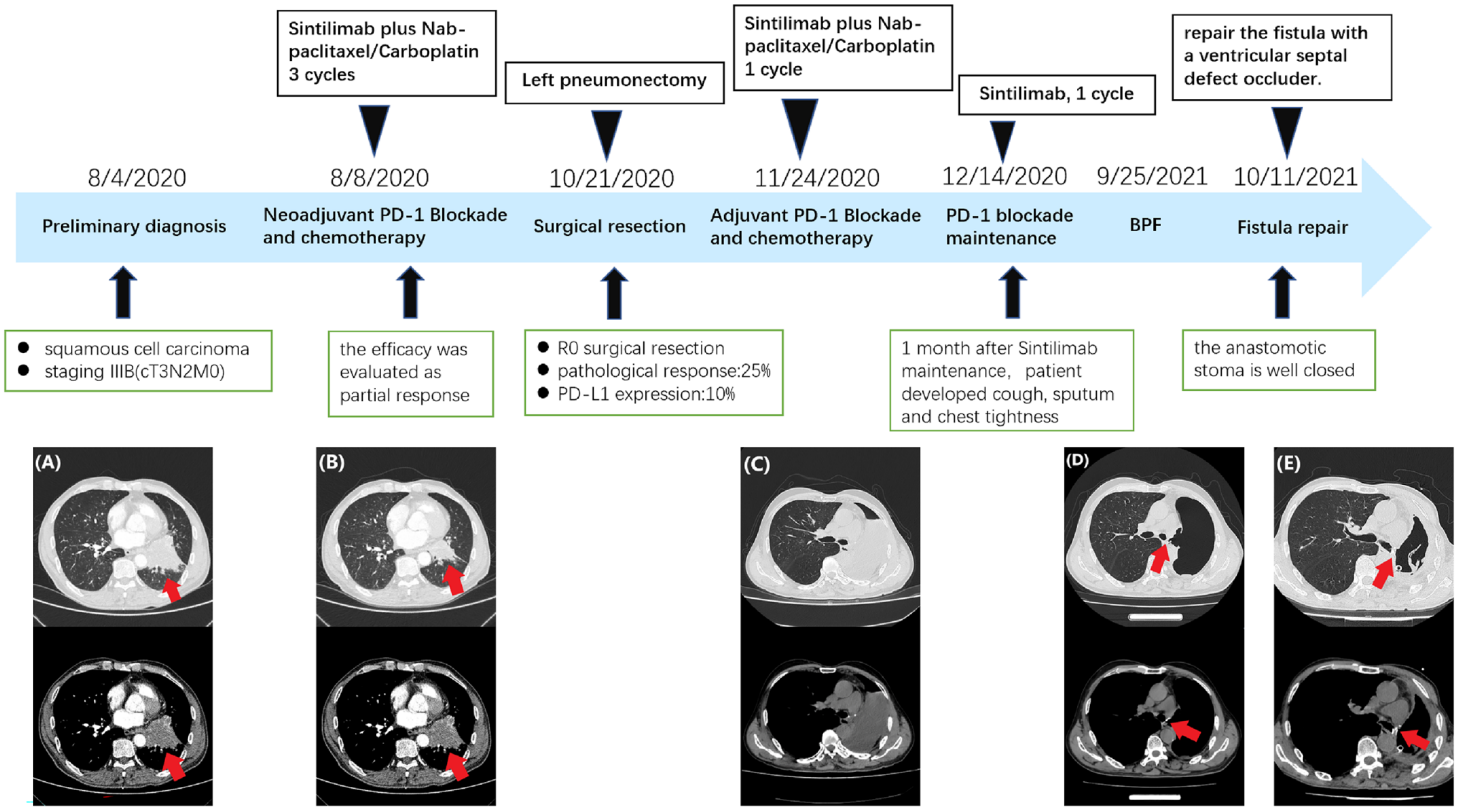
The timeline of the patient’s treatment course and evaluation of imaging efficacy. **A** Chest CT image before neoadjuvant therapy on 5 August 2020 shows a high-density shadow in the left hilum, about 6.0 × 5.7 cm in size (arrow). **B** Chest CT image obtained on 11 October 2020 after three cycles of neoadjuvant therapy shows that the left hilar mass was significantly reduced compared to before (arrow). **C** Chest CT image after 1 month of left pneumonectomy on 23 November 2020. **D** Chest CT image on 25 September 2021 shows a fistulous tract of the left main bronchus (arrow). **E** Chest CT image obtained on 3 November 2021 shows that the fistulous tract is well closed (arrow)

**Fig. 4:**
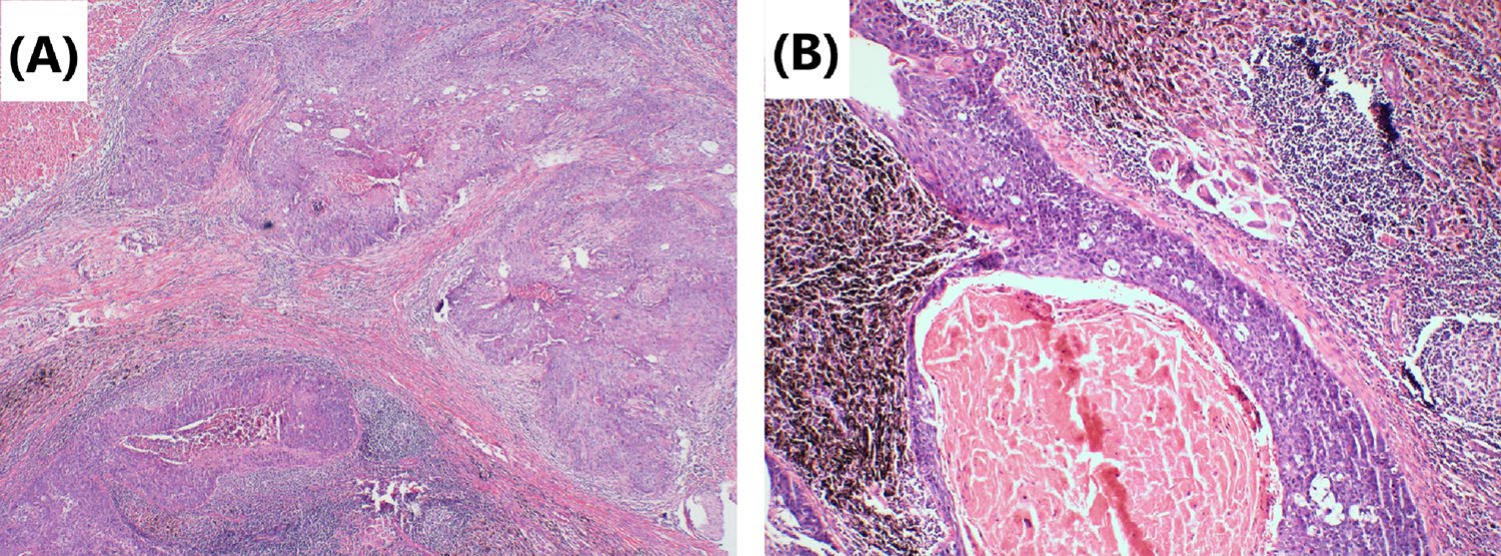
Tumor cells’ morphology is shown by hematoxylin–eosin staining. **A** The primary tumor is mainly accompanied by immune infiltrating changes, accompanied by tumor necrotic cells after neoadjuvant immunochemotherapy. There were more lymphocyte infiltration around the cancer nest and the interstitial fibrous tissue hyperplasia. Original magnification × 40. **B** Lymph-node metastases are mainly necrotic and repairing changes after neoadjuvant immunochemotherapy can be observed. Fibrous tissue hyperplasia around the cancer nest with multinucleated giant cell reaction. Original magnification × 200

A 59-year-old male patient diagnosed with stage IIIB (cT3N2M0) squamous cell lung carcinoma presented to our hospital. The timeline of the patient’s course of treatment and the evaluation of imaging efficacy are described in detail in Fig. 3. The patient underwent three cycles of neoadjuvant immunochemotherapy and stopped medication 33 days prior to radical surgery. The therapeutic effect was evaluated as PR. A left pneumonectomy was performed by video-assisted thoracoscopy. The postoperative pathological changes are shown in Fig. 4. The patient received one cycle of adjuvant immunochemotherapy 34 days after the operation and one cycle of single sintilimab maintenance treatment. Approximately 1 month after sintilimab maintenance treatment, he developed a cough, sputum, and chest tightness. After 2 weeks of oral antibiotics, the above symptoms were alleviated. The bronchoscopy, which was performed on 5 March 2021, did not reveal a fistula. In September 2021, symptoms such as cough and sputum recurred. Re-examination of chest CT revealed a linear low-density shadow connection between the left main bronchus and the pleural cavity (Fig. 3D). Bronchoscopy revealed a fistula with a diameter of about 3 mm in the left main bronchus. After fully discussing the risks and benefits of surgery and the limited options available, we attempted to repair the fistula with a ventricular septal defect occluder. At present, a patient has been followed up for more than 1 year without receiving any maintenance treatment. Chest CT scans have been reviewed every 3 months without recurrence of lung cancer. On 3 November 2021, chest CT showed that the fistulous tract was fully closed (Fig. 3E).

## Case 3

**Fig. 5:**
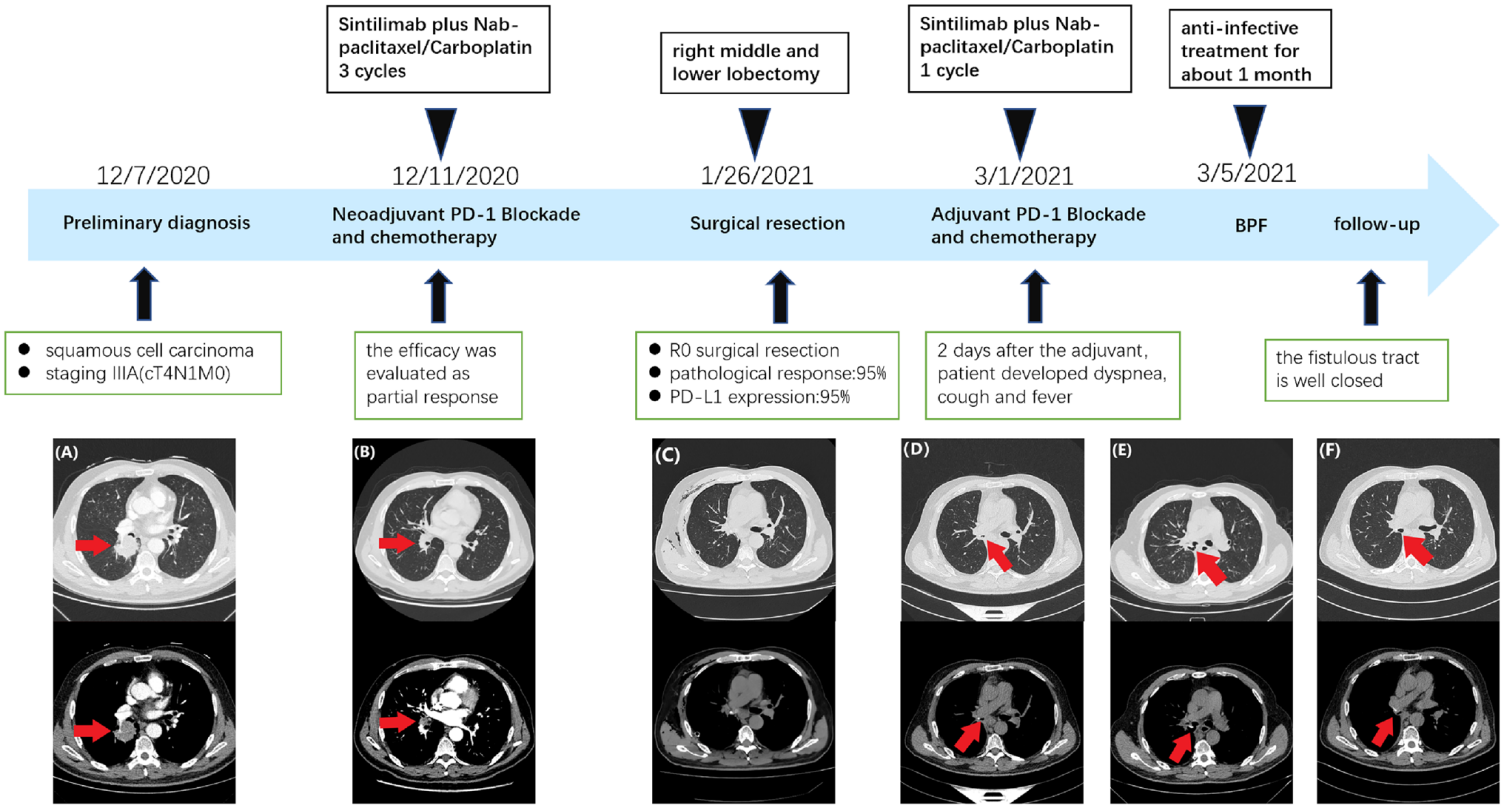
The timeline of the patient’s treatment course and evaluation of imaging efficacy. **A** Chest CT image obtained on 7 December 2020 before neoadjuvant therapy shows an irregular high-density shadow in the right lower lobe, about 3.9 × 3.4 cm in size (arrow). **B** Chest CT image obtained on 20 January 2021 after three cycles of neoadjuvant therapy shows that the mass shadow in the right lower lobe was significantly reduced compared to before (arrow). **C** Chest CT image after 5 days of right middle and lower lobectomy on 31 January 2021. **D** Chest CT image 1 day before adjuvant therapy shows no obvious abnormality on 1 March 2021. **E** Chest CT image on 5 March 2021 shows a fistulous tract of the right main bronchus (arrow). **F** Chest CT image obtained on 31 August 2021 shows that the fistulous tract is well closed (arrow)

**Fig. 6:**
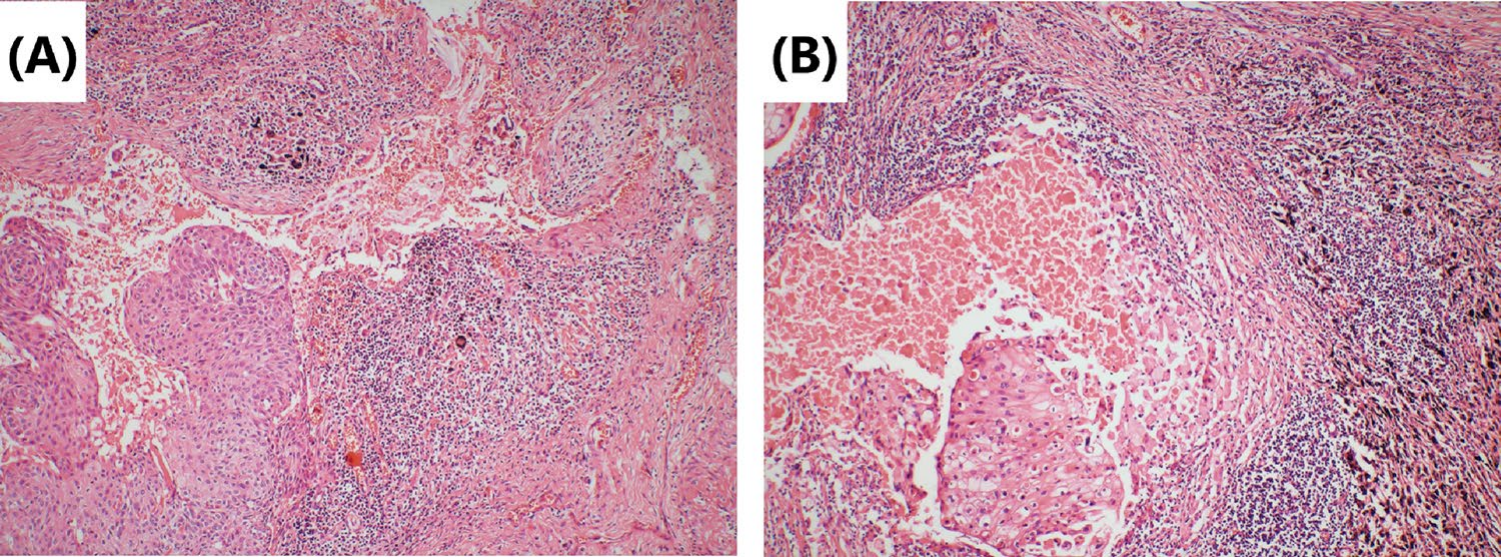
Tumor cells’ morphology is shown by hematoxylin–eosin staining. **A** The primary tumor mainly shows immune infiltrating and necrotic changes after neoadjuvant immunochemotherapy. A large number of lymphocytes infiltrated the cancer nest, and there was a multinucleated giant cell reaction. Original magnification × 100. **B** Lymph-node metastases mainly include necrotic changes after neoadjuvant immunochemotherapy. There are more foamy histiocytic infiltration and fibrous tissue proliferation around the cancer nest. Original magnification × 200

A 62-year-old male patient diagnosed with stage IIIA (cT4N1M0) squamous cell lung carcinoma presented to our hospital. The patient’s treatment course and imaging response assessment are described in detail in Fig. 5. The patient underwent two cycles of neoadjuvant immunochemotherapy, and the therapeutic effect was evaluated as PR. Right middle and lower lobectomy were performed by video-assisted thoracoscopy 25 days after neoadjuvant therapy. The postoperative pathological changes are shown in Fig. 6. Chest CT image before adjuvant therapy showed no obvious abnormalities (Fig. 5D). Also, the patient received adjuvant therapy 35 days after the operation. However, the patient developed dyspnea, cough, and fever (highest temperature was 38.3 ℃, grade 1) 2 days after the first cycle of adjuvant therapy. Chest CT showed a gas density shadow in the right thoracic cavity connected to the bronchial stump, a sign of a bronchopleural fistula (Fig. 5E). After anti-infective treatment for about 1 week, his symptoms were significantly relieved. On 31 August 2021, no fistula was observed on the chest CT (Fig. 5F). Chest CT scans were reviewed every 3 months, and there was no recurrence of lung cancer. At present, the patient is still undergoing continuous follow-up without maintenance treatment.

## Case 4

**Fig. 7:**
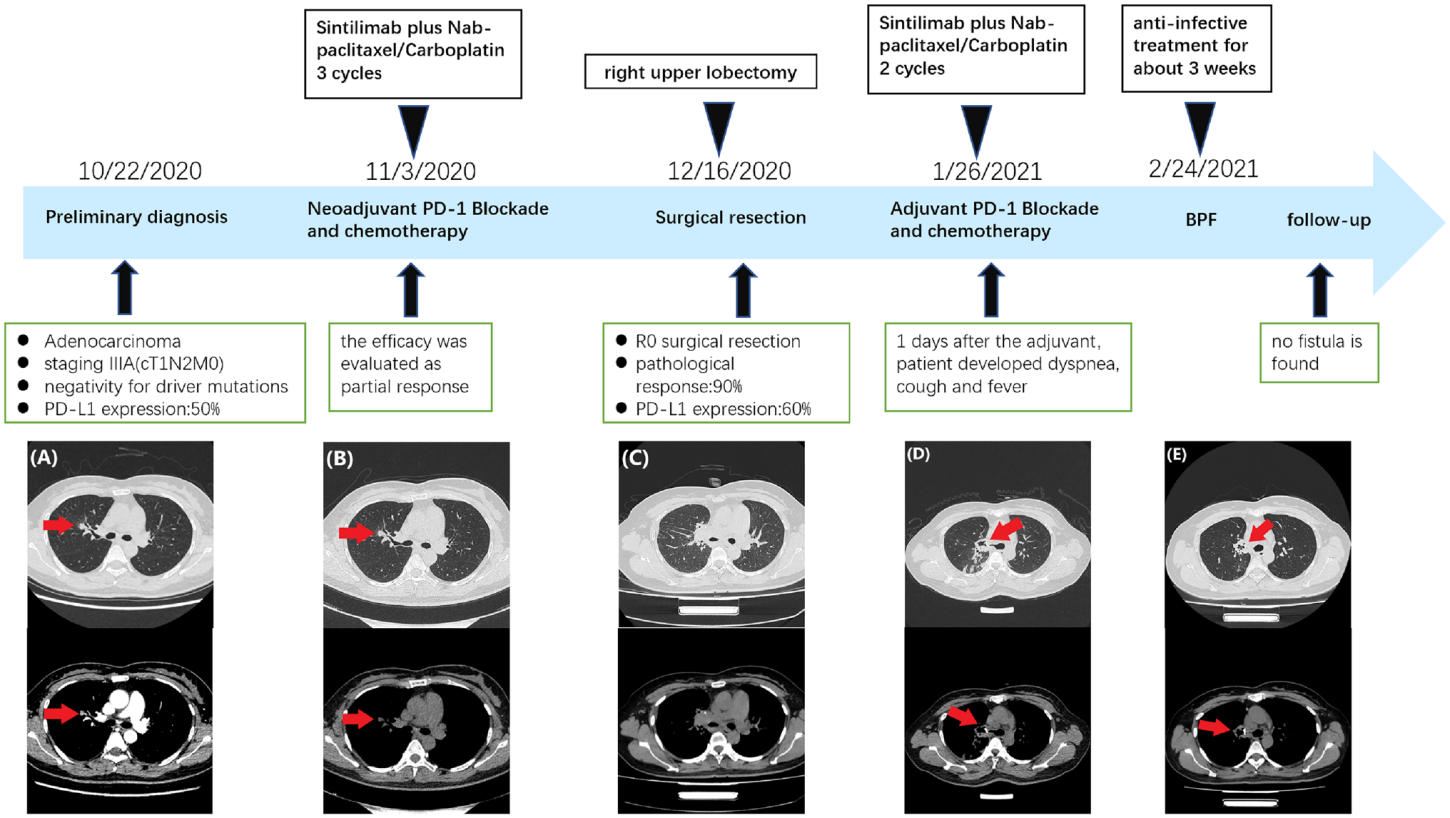
The timeline of the patient’s treatment course and evaluation of imaging efficacy. **A** Chest CT image obtained on 3 November 2020 before neoadjuvant therapy shows an irregular nodule in the upper lobe of the right lung, about 0.8 cm in size (arrow). **B** Chest CT image obtained on 14 December 2020 after three cycles of neoadjuvant therapy shows no significant change in the right upper lobe nodule compared to the previous (arrow). **C** Chest CT image obtained on 22 December 2020 after 6 days of right upper lobectomy. **D** Chest CT image obtained on 3 March 2021 shows irregular gas density on the right side of the bronchial stump (arrow). **E** Chest CT image obtained on 14 July 2021 shows that the gas density shadow on the right side of the bronchial stump was smaller than before, and no fistula was found between the main bronchus and the pleural cavity (arrow)

**Fig. 8:**
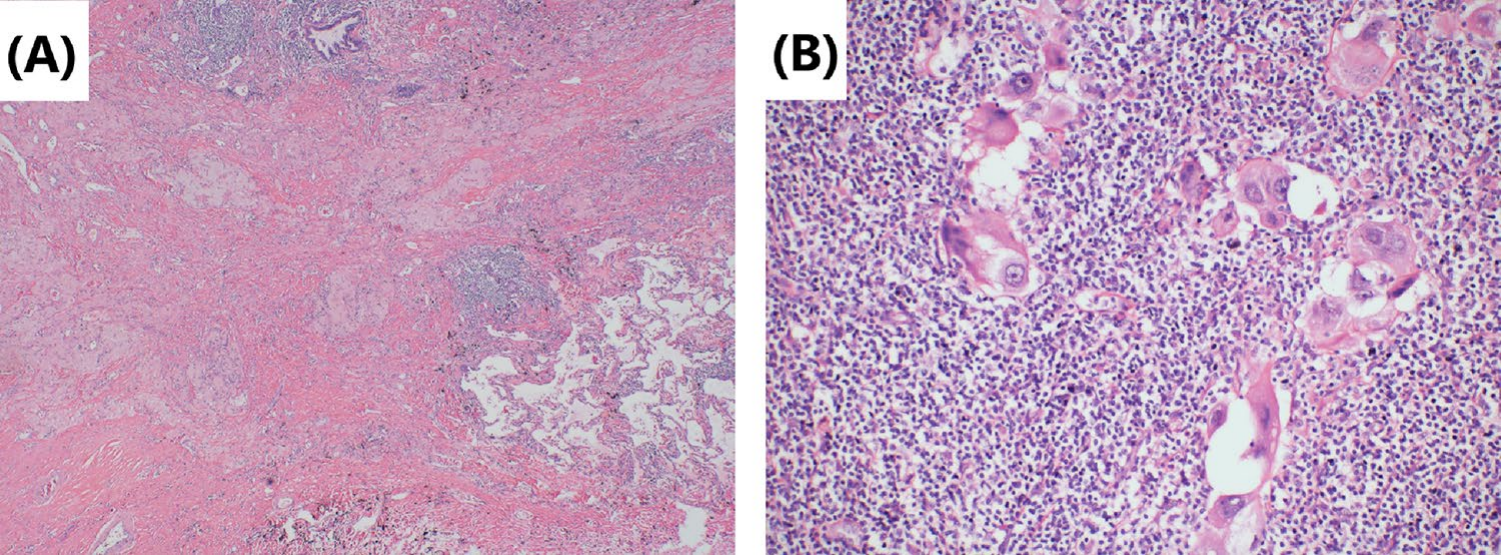
Tumor cells’ morphology is shown by hematoxylin–eosin staining. **A** Repairing changes are seen after neoadjuvant immunochemotherapy. Fibrous tissue hyperplasia in the tumor bed and peripheral focal lymphocytic infiltration. Original magnification × 40. **B** The cancer cells in the lymph-node metastasis are significantly degenerated, and the peripheral lymphocytes are infiltrated. There are also no obvious repairing and necrosis changes after neoadjuvant immunochemotherapy. Original magnification × 200

A 43-year-old female patient diagnosed with stage IIIA (cT1N2M0) lung adenocarcinoma presented to our hospital. Her driver mutations (EGFR/ALK/KRAS/RET/ROS1) were negative. The patient’s treatment process and evaluation of imaging efficacy are shown in detail in Fig. 7. The patient underwent 2 cycles of neoadjuvant immunochemotherapy, and the therapeutic effect was evaluated as PR. A right upper lobectomy was performed by video-assisted thoracoscopy 22 days after neoadjuvant therapy. The postoperative pathological changes are shown in Fig. 8. The patient received 2 cycles of adjuvant therapy 42 days after the operation. One day after the second cycle of adjuvant therapy, she developed a fever (highest temperature was 38.8 ℃, grade 1). Chest CT image shows irregular gas density in the right thoracic cavity, a sign of a fistula in the bronchial stump (Fig. 7D). After 3 weeks of anti-infective treatment, the patient’s symptoms were gradually relieved. Chest CT image obtained on 14 July 2021 shows that the gas density shadow on the right side of the bronchial stump was smaller than before, and no fistula was found between the main bronchus and the pleural cavity (Fig. 7E). Chest CT scans were reviewed every 3 months without recurrence of lung cancer. At present, a patient has been followed up for more than 1 year without maintenance treatment.

## Discussion

Over recent years, the use of immune checkpoint inhibitors for treating patients with resectable NSCLC has been receiving increasing interest. Clinical studies of neoadjuvant immunotherapy show that the incidence of postoperative BPF is approximately 2–9% (Cascone et al. 2021; Dai et al. 2022; Wislez et al. 2022). In the IONESCO study (Wislez et al. 2022), 43 patients underwent surgery after receiving 3 cycles of durvalumab immune monotherapy, and 1 patient died due to postoperative BPF. The interim results of the NEOSTAR study (Cascone et al. 2019) revealed that among 23 NSCLC patients who received 2 cycles of single-agent Nivolumab before surgery, 2 patients developed BPF postoperatively, with 1 of them eventually succumbing to secondary pulmonary infection. A retrospective neoadjuvant study (Dai et al. 2022) of immune combination chemotherapy conducted by Dai et al. reported that 1 out of 23 patients who underwent surgery developed BPF on the 12th postoperative day. Despite attempts at bronchoscopic fistula repair, this patient ultimately succumbed to severe infection at the anastomotic site. However, BPF is a serious and fatal complication after pulmonary resection that is very rarely reported, especially in patients receiving neoadjuvant immunotherapy or immunochemotherapy. As it is often confined to a single case, only four cases have been reported in the previous literature (Cao et al. 2020; Menezes et al. 2020; Cascone et al. 2021; Dai et al. 2022). Among these cases, the information about BPF was very limited for three patients, while there was only one case with a detailed description of BPF. The patient was diagnosed with lung adenocarcinoma (cT2bN2M0, stage IIIA), and he accepted radical surgery after three cycles of combination neoadjuvant therapy of pembrolizumab plus cisplatin and pemetrexed. Three weeks later, he developed cough and fever, and after undergoing a bronchoscopy and lung CT scan, he was diagnosed with BPF. A drainage chest tube was immediately placed, normal saline irrigation and antibiotic therapy were applied twice a day, and his fistula gradually closed after 2 months (Cao et al. 2020).

In the present study, patients were predominantly male, 59 ± 8 years old, smokers, treated with neoadjuvant immunochemotherapy, and mostly with central type lung cancer with stage III. The baseline characteristics of all patients (*n* = 8) are presented in Table 1. To the best of our knowledge, this is the first study that systematically described the clinical process, postoperative pathological changes, as well as risk factors of BPF patients treated with neoadjuvant immunotherapy or immunochemotherapy. As the number of BPFs was low, it was not possible to perform an analysis of risk factors for the BPF.

**Table 1:** Demographic and clinical characteristics of the patients

	ECOG	Smoking	Diagnosis	PD-L1^a^	PD-L1^b^	Location	Clinical stage	Neoadjuvant therapy	Cycles	Evaluable lesions	RECIST	Operation time^c^	Operation	BPF occurrence^d^	PRR	CTCAE	Outcomes
Case 1	0	Former	SqCC	NA	< 1%	LL + Central	T4N1M0 IIIA	TC + sintilimab	3	− 40%	PR	25 days	LP	44 days	80%	5	Death
Case 2	0	Former	SqCC	< 1%	10%	LL + Central	T3N2M0 IIIB	TC + sintilimab	3	− 38%	PR	33 days	LP	103 days	25%	3	Recover
Case 3	0	Current	SqCC	80%	95%	RL + Central	T4N1M0 IIIA	TC + sintilimab	2	− 41%	PR	25 days	RML + RLL	37 days	95%	2	Recover
Case 4	0	Never	AD	50%	60%	RL + Peripheral	T1N2M0 IIIA	TC + sintilimab	2	− 40%	PR	22 days	RUL	69 days	90%	2	Recover
Case 5 (Cao et al. 2020)	NA	NA	AD	NA	NA	RL + NA	T2bN2M0 IIIA	AP + pembrolizumab	3	NA	SD	14 days	RUL	21 days	NA	3	Recover
Case 6 (Dai et al. 2022)	1	Current	AD	NA	NA	RL + Central	T2N2M0 IIIA	TC + pembrolizumab + VEGFR	4	NA	PR	NA	RUL + DS	11 days	NA	5	Death
Case 7 (Cascone et al. 2021)	NA	NA	NA	NA	NA	NA	TxNxM0I–IIIA	nivolumab	3	NA	NA	NA	NA	NA	NA	5	Death
Case 8 (Menezes et al. 2020)	NA	Never	NSCLC	NA	NA	LL + Central	NA	neoadjuvant immunotherapy	NA	NA	NA	NA	LP	2 months	NA	3	Recover

*ECOG* Eastern Cooperative Oncology Group score, *NA* not available, *SqCC* squamous cell carcinoma, *AD* adenocarcinoma, *TPS* tumor proportion score, *LL* left lung, *RL* right lung, *T* nab-paclitaxel, *C* carboplatin, *P* cisplatin, *A* pemetrexed, *VEGFR* vascular endothelial growth factor receptor including bevacizumab and apatinib, *RECIST* Response Evaluation Criteria In Solid Tumors 1.1, *PR* partial response, *SD* stable disease, *LP* left pneumonectomy, *RP* right pneumonectomy, *RML* right middle lobectomy, *RLL* right lower lobectomy, *RUL* right upper lobectomy, *DS* dorsal segmentectomy, *PRR* Pathological Remission Rate, *CTCAE* Common Terminology Criteria for Adverse Events 5.0

**a**, PD-L1 of baseline. **b**, PD-L1 of postoperative specimens. **c**, The operation time is counted from the first day of the last cycle of neoadjuvant therapy. **d**, Time from surgery to patient onset of typical symptoms of BPF

Over recent years, increasing data have been emerging on the beneficial effect of using ICIs for the neoadjuvant therapy of patients with early stage (IB, II, and resectable III) NSCLC (Yang et al. 2018b; Provencio et al. 2020; Cascone et al. 2021). BPF is one of the most serious postoperative adverse events and a potentially fatal complication of pulmonary resection. Several studies revealed that pneumonectomy is a high-risk surgical procedure, and the incidence of BPF is higher than general lobectomy (Asamura et al. 1992; Dancewicz et al. 2006; Brunelli et al. 2020). In addition, the incidence of BPF after right-sided pneumonectomy is higher than that after left-sided pneumonectomy (Wright et al. 1996; Deschamps et al. 2001; Algar et al. 2001; Darling et al. 2005; Brunelli et al. 2020). In the present study, a detailed description of the operation was available for seven patients, where three accepted left pneumonectomy, and four patients adopted lobectomy. These results were not consistent with the previous reports, which may be due to the limited number of patients.

Although surgery is suspected to be implicated in the BPF study’s development, we could not completely rule out immunotherapy or immunochemotherapy. First, several reports (Rendina et al. 1997; Pataer et al. 2012; Weissferdt et al. 2020) showed that potential healing barriers to reconstructed bronchi due to diffuse fibrotic responses, necrosis, lack of interstitial space, inflammation in the tumor specimens, and impaired vascularization increase the difficulty of surgical resection and may increase the incidence of perioperative complications. Also, neoadjuvant chemotherapy was formerly considered a risk factor for BPF (Hu et al. 2013; Ayten et al. 2021). In this study, we summarized the characteristics of the pathological changes after operation, where 50% (2/4) of resected tumors showed a major pathologic response (MPR) (Case 1 and Case 4), reparative changes were mainly in the tumor bed, and the fibrous tissue around the cancer nest was significantly proliferated, accompanied by chronic inflammatory cell or lymphocyte infiltration (Figs. 2 and 8). In the other 2 patients (Case 2 and Case 3), the tumor bed was dominated by immune infiltrative changes, which were accompanied by necrosis, and a large quantity of lymphocytes infiltrated around the tumor nest (Figs. 4 and 6). These characteristics of cell death and repair changes are consistent with the previous literatures (Cottrell et al. 2018; Liang et al. 2021), suggesting that the pathological specimens of patients receiving neoadjuvant immunochemotherapy include more vascular elastic fiber destruction, vascular wall degeneration, fibrinoid necrosis and fibrosis, and pulmonary interstitial exudation compared with neoadjuvant chemotherapy patients (Liang et al. 2021). Consequently, it may be more difficult to perform complex reconstruction operations after preoperative treatment added with PD-1 inhibitors, and patients are more prone to postoperative complications such as BPF or prolonged air leak (Yang et al. 2018a). Second, postoperative BPF can be classified as acute, subacute, and chronic, where the former is typically described after surgery. BPFs are most commonly described in an acute form (Lois and Noppen 2005), which is consistent with the study by Mammana et al. (Mammana et al. 2020) who reported that among the 23 BPF NSCLC patients who underwent pneumonectomy, 19 (82.6%) had early BPF and 4 (17.4%) had late BPF (occurring after 30 days). In comparison, it should be noted that postoperative BPF after immunotherapy or immunochemotherapy may occur in the late stage (5/7, 71.4%). As shown in Table 1, there were 2 cases with BPF 1 month later and 3 cases with BPF 2 months later. In summary, these observations may not necessarily result from the operation, as BPF or fistular was also described in patients with advanced stages of NSCLC and other types of cancer who did not undergo operation. Nowadays, durvalumab consolidation is considered a standard treatment for most patients with stage III NSCLC after chemoradiotherapy. A patient with stage IIIB NSCLC developed bronchomediastinal fistula, which was reported after 3 cycles of durvalumab consolidation (Sumi et al. 2019). Although the possibility of chemoradiation-induced necrosis cannot be completely ruled out, effective durvalumab therapy was more likely associated with the mucosal defect, contributing to the development of bronchomediastinal fistula. In addition, BPF can occur even after 10 months of durvalumab consolidation (Darwin et al. 2021), which demonstrates that immunotherapy is most likely related to the development of BPF. Moreover, the study by Ashley et al. suggests that preoperative immunotherapy in patients with advanced head and neck cancer may lead to delayed wound healing (Mays et al. 2021). Therefore, it is possible that neoadjuvant and adjuvant immunochemotherapy may delay wound healing and increase the chance of tissue defect, contributing to BPF.

Based on the analysis above, it is worth noting that the timing of surgery may also be a risk factor for BPF. Indeed, previous studies reported that the earlier timing of surgery might contribute to the occurrence of BPF (Hao et al. 2021). In this study (Table 1), the surgery in most patients was performed within 30 days after the start of the last cycle of neoadjuvant therapy. In the NADIM study, surgery was planned to be performed 42–49 days after the start of the third treatment cycle (Provencio et al. 2020). Also, in the NEOSTAR study, the median time to surgical resection was 31 days after the last dose of nivolumab (range 21–87 days) (Cascone et al. 2021). Therefore, the association between the timing of surgery and the incidence of BPF should be further investigated in the future.

In this study, eight patients developed BPF, and the overall mortality in these eight patients was 37.5 percent. Furthermore, with the growing application of ICIs in neoadjuvant therapy for NSCLC patients, the number of BPF may increase. Therefore, it is important to reduce the incidence of BPF, which remains a great challenge for thoracic surgeons. As with most postoperative complications, prevention is essential for management, especially for patients with a high risk of BPF. Several studies reported that reinforcing the bronchial stump with a pericardial flap or intercostal muscle flap is a safe and effective method for preventing BPF (Taghavi et al. 2005; Sfyridis et al. 2007; Barbetakis et al. 2008; Matsuoka et al. 2016). However, in the cases we reported, the method of strengthening the bronchial stump was not employed to prevent the occurrence of BPF. In the present study, the primary tumor location of five out of six patients was a central type, and there were six patients with definite stage information, all stage IIIA. Based on these findings, these factors may be related to BPF in patients with neoadjuvant immunochemotherapy, which should be further studied. In addition, perioperative management is also important to reduce the incidence of BPF. For example, it is necessary to actively control blood glucose, perform anti-infection, correct hypoalbuminemia, and minimize the use of steroids before surgery (Chu 2018). Relevant studies (Guo and Dipietro 2010) have reported that elevated blood glucose levels or systemic steroid use are risk factors for delayed wound healing, but none of the four patients we reported had used steroids. Although Case 3 has elevated fasting blood glucose, it does not meet the diagnostic criteria for diabetes. Timely and prompt removal of the closed chest drainage tube and tracheal intubation after surgery (Sirbu et al. 2001) in patients who accept neoadjuvant immunochemotherapy with stage III central type lung cancer is also of utmost importance.

In conclusion, although BPF is rare in patients receiving neoadjuvant immunochemotherapy, more attention should be paid to this issue because of its life-threatening characteristics. Nevertheless, the present study improves our understanding of neoadjuvant immunochemotherapy. Therefore, perhaps, reinforcement of the bronchial stump should be recommended in cases of neoadjuvant immunochemotherapy for lung cancer patients, especially for those with a higher risk of BPF, including central type lung cancer of stage III.

## Data Availability

All data generated or analyzed during this study are included in this published article. Data sharing is not applicable to this article as no datasets were generated or analyzed during the current study.

## Declarations

### Provenance and peer review

Not commissioned; externally peer reviewed.

## References

[CR1] Algar FJ, Alvarez A, Aranda JL et al (2001) Prediction of early bronchopleural fistula after pneumonectomy: a multivariate analysis. Ann Thorac Surg 72:1662–1667. 10.1016/s0003-4975(01)03096-x 11722062 10.1016/s0003-4975(01)03096-x

[CR2] Asamura H, Naruke T, Tsuchiya R et al (1992) Bronchopleural fistulas associated with lung cancer operations. Univariate and multivariate analysis of risk factors, management, and outcome. J Thorac Cardiovasc Surg 104:1456–1464 1434730

[CR3] Ayten O, Ozdemir C, Sokucu SN et al (2021) The role of interventional pulmonology for the postoperative bronchopleural fistula. Niger J Clin Pract 24:633–639. 10.4103/njcp.njcp_614_19 34018970 10.4103/njcp.njcp_614_19

[CR4] Barbetakis N, Samanidis G, Tsilikas C (2008) eComment: Pedicled pericardial flap for prevention of postpneumonectomy bronchopleural fistula. A safe alternative. Interact Cardiovasc Thorac Surg 7:642. 10.1510/icvts.2008.177782C 18664667 10.1510/icvts.2008.177782C

[CR5] Brunelli A, Rocco G, Szanto Z et al (2020) Morbidity and mortality of lobectomy or pneumonectomy after neoadjuvant treatment: an analysis from the ESTS database. Eur J Cardiothorac Surg 57:740–746. 10.1093/ejcts/ezz287 31638692 10.1093/ejcts/ezz287 PMC7825477

[CR6] Cao M, Fu Y, Xiao X et al (2020) Bronchopleural fistula following a video-assisted thoracoscopic surgery lobectomy after neoadjuvant therapy of pembrolizumab: a case report and literature review. Ann Transl Med 8:1691. 10.21037/atm-20-7582 33490203 10.21037/atm-20-7582 PMC7812198

[CR7] Cascone T, William WN, Weissferdt A et al (2019) Neoadjuvant nivolumab (N) or nivolumab plus ipilimumab (NI) for resectable non-small cell lung cancer (NSCLC): clinical and correlative results from the NEOSTAR study. J Clin Oncol. 10.1200/JCO.2019.37.15_suppl.8504

[CR8] Cascone T, William WN, Weissferdt A et al (2021) Neoadjuvant nivolumab or nivolumab plus ipilimumab in operable non-small cell lung cancer: the phase 2 randomized NEOSTAR trial. Nat Med 27:504–514. 10.1038/s41591-020-01224-2 33603241 10.1038/s41591-020-01224-2 PMC8818318

[CR9] Chu X (2018) Bronchopleural fistula—the pulmonary surgery complications that should be emphasized by thoracic surgeons. Zhongguo Fei Ai Za Zhi 21:239–240. 10.3779/j.issn.1009-3419.2018.03.27 29587952 10.3779/j.issn.1009-3419.2018.03.27 PMC5973032

[CR10] Cottrell TR, Thompson ED, Forde PM et al (2018) Pathologic features of response to neoadjuvant anti-PD-1 in resected non-small-cell lung carcinoma: a proposal for quantitative immune-related pathologic response criteria (irPRC). Ann Oncol 29:1853–1860. 10.1093/annonc/mdy218 29982279 10.1093/annonc/mdy218 PMC6096736

[CR11] Dai J, Zhu X, Li D et al (2022) Sleeve resection after neoadjuvant chemoimmunotherapy in the treatment of locally advanced non-small cell lung cancer. Transl Lung Cancer Res 11:188–200. 10.21037/tlcr-22-56 35280313 10.21037/tlcr-22-56 PMC8902091

[CR12] Dancewicz M, Kowalewski J, Peplinski J (2006) Factors associated with perioperative complications after pneumonectomy for primary carcinoma of the lung. Interact Cardiovasc Thorac Surg 5:97–100. 10.1510/icvts.2005.118125 17670525 10.1510/icvts.2005.118125

[CR13] Darling GE, Abdurahman A, Yi Q-L et al (2005) Risk of a right pneumonectomy: role of bronchopleural fistula. Ann Thorac Surg 79:433–437. 10.1016/j.athoracsur.2004.07.009 15680809 10.1016/j.athoracsur.2004.07.009

[CR14] Darwin A, Rose T, Tandon A, Tanvetyanon T (2021) Development of bronchopleural fistula after durvalumab consolidation for stage III non-small-cell lung cancer. Clin Lung Cancer 22:e18–e24. 10.1016/j.cllc.2020.07.010 32828661 10.1016/j.cllc.2020.07.010

[CR15] Deschamps C, Bernard A, Nichols FC et al (2001) Empyema and bronchopleural fistula after pneumonectomy: factors affecting incidence. Ann Thorac Surg 72:243–247. 10.1016/s0003-4975(01)02681-9. (**discussion 248**) 11465187 10.1016/s0003-4975(01)02681-9

[CR16] Doroshow DB, Sanmamed MF, Hastings K et al (2019) Immunotherapy in non-small cell lung cancer: facts and hopes. Clin Cancer Res 25:4592–4602. 10.1158/1078-0432.CCR-18-1538 30824587 10.1158/1078-0432.CCR-18-1538 PMC6679805

[CR17] Guo S, Dipietro LA (2010) Factors affecting wound healing. J Dent Res 89:219–229. 10.1177/0022034509359125 20139336 10.1177/0022034509359125 PMC2903966

[CR18] Hao L, Hu Y, Hu J et al (2021) Case report: a squamous cell lung carcinoma patient who responded to neoadjuvant immunochemotherapy but died from anastomosis leakage or/and irAEs: immune microenvironment and genomic features changes. Front Oncol 11:674328. 10.3389/fonc.2021.674328 34367960 10.3389/fonc.2021.674328 PMC8339907

[CR19] Hu X, Duan L, Jiang G et al (2013) A clinical risk model for the evaluation of bronchopleural fistula in non-small cell lung cancer after pneumonectomy. Ann Thorac Surg 96:419–424. 10.1016/j.athoracsur.2013.04.050 23782644 10.1016/j.athoracsur.2013.04.050

[CR20] Liang H, Yang C, Gonzalez-Rivas D et al (2021) Sleeve lobectomy after neoadjuvant chemoimmunotherapy/chemotherapy for local advanced non-small cell lung cancer. Transl Lung Cancer Res 10:143–155. 10.21037/tlcr-20-778 33569300 10.21037/tlcr-20-778 PMC7867787

[CR21] Lois M, Noppen M (2005) Bronchopleural fistulas: an overview of the problem with special focus on endoscopic management. Chest 128:3955–3965. 10.1378/chest.128.6.3955 16354867 10.1378/chest.128.6.3955

[CR22] Mammana M, Marulli G, Zuin A et al (2020) Postpneumonectomy bronchopleural fistula: analysis of risk factors and the role of bronchial stump coverage. Surg Today 50:114–122. 10.1007/s00595-019-01871-0 31493198 10.1007/s00595-019-01871-0

[CR23] Matsuoka K, Imanishi N, Yamada T et al (2016) Clinical results of bronchial stump coverage using free pericardial fat pad. Interact Cardiovasc Thorac Surg 23:553–559. 10.1093/icvts/ivw193 27338871 10.1093/icvts/ivw193

[CR24] Mays AC, Yarlagadda B, Achim V et al (2021) Examining the relationship of immunotherapy and wound complications following flap reconstruction in patients with head and neck cancer. Head Neck 43:1509–1520. 10.1002/hed.26601 33417293 10.1002/hed.26601 PMC8893989

[CR25] Menezes V, Soder S, Kadadah S et al (2020) Bronchoscopic treatment of a bronchopleural fistula after pneumonectomy. JTCVS Tech 4:345–348. 10.1016/j.xjtc.2020.08.016 34318070 10.1016/j.xjtc.2020.08.016 PMC8303052

[CR26] Pataer A, Kalhor N, Correa AM et al (2012) Histopathologic response criteria predict survival of patients with resected lung cancer after neoadjuvant chemotherapy. J Thorac Oncol 7:825–832. 10.1097/JTO.0b013e318247504a 22481232 10.1097/JTO.0b013e318247504a PMC3465940

[CR27] Provencio M, Nadal E, Insa A et al (2020) Neoadjuvant chemotherapy and nivolumab in resectable non-small-cell lung cancer (NADIM): an open-label, multicentre, single-arm, phase 2 trial. Lancet Oncol 21:1413–1422. 10.1016/S1470-2045(20)30453-8 32979984 10.1016/S1470-2045(20)30453-8

[CR28] Rendina EA, Venuta F, De Giacomo T et al (1997) Safety and efficacy of bronchovascular reconstruction after induction chemotherapy for lung cancer. J Thorac Cardiovasc Surg 114:830–835. 10.1016/S0022-5223(97)70088-6. (**discussion 835–837**) 9375614 10.1016/S0022-5223(97)70088-6

[CR29] Sfyridis PG, Kapetanakis EI, Baltayiannis NE et al (2007) Bronchial stump buttressing with an intercostal muscle flap in diabetic patients. Ann Thorac Surg 84:967–971. 10.1016/j.athoracsur.2007.02.088 17720409 10.1016/j.athoracsur.2007.02.088

[CR30] Sirbu H, Busch T, Aleksic I et al (2001) Bronchopleural fistula in the surgery of non-small cell lung cancer: incidence, risk factors, and management. Ann Thorac Cardiovasc Surg 7:330–336 11888471

[CR31] Sumi T, Ikeda T, Kure K et al (2019) Bronchomediastinal fistula during durvalumab therapy after chemoradiotherapy in stage III NSCLC. J Thorac Oncol 14:1860–1861. 10.1016/j.jtho.2019.06.003 31300340 10.1016/j.jtho.2019.06.003

[CR32] Taghavi S, Marta GM, Lang G et al (2005) Bronchial stump coverage with a pedicled pericardial flap: an effective method for prevention of postpneumonectomy bronchopleural fistula. Ann Thorac Surg 79:284–288. 10.1016/j.athoracsur.2004.06.108 15620959 10.1016/j.athoracsur.2004.06.108

[CR33] Weissferdt A, Pataer A, Vaporciyan AA et al (2020) Agreement on major pathological response in NSCLC patients receiving neoadjuvant chemotherapy. Clin Lung Cancer 21:341–348. 10.1016/j.cllc.2019.11.003 32279936 10.1016/j.cllc.2019.11.003 PMC7305995

[CR34] Wislez M, Mazieres J, Lavole A et al (2022) Neoadjuvant durvalumab for resectable non-small-cell lung cancer (NSCLC): results from a multicenter study (IFCT-1601 IONESCO). J Immunother Cancer 10:e005636. 10.1136/jitc-2022-005636 36270733 10.1136/jitc-2022-005636 PMC9594538

[CR35] Wright CD, Wain JC, Mathisen DJ, Grillo HC (1996) Postpneumonectomy bronchopleural fistula after sutured bronchial closure: incidence, risk factors, and management. J Thorac Cardiovasc Surg 112:1367–1371. 10.1016/S0022-5223(96)70153-8 8911336 10.1016/S0022-5223(96)70153-8

[CR36] Yang C-FJ, McSherry F, Mayne NR et al (2018a) Surgical outcomes after neoadjuvant chemotherapy and ipilimumab for non-small cell lung cancer. Ann Thorac Surg 105:924–929. 10.1016/j.athoracsur.2017.09.030 29258674 10.1016/j.athoracsur.2017.09.030

[CR37] Yang X, Yin R, Xu L (2018b) Neoadjuvant PD-1 blockade in resectable lung cancer. N Engl J Med 379:e14. 10.1056/NEJMc1808251 30179394 10.1056/NEJMc1808251

